# Methods Used to Evaluate Pain Behaviors in Rodents

**DOI:** 10.3389/fnmol.2017.00284

**Published:** 2017-09-06

**Authors:** Jennifer R. Deuis, Lucie S. Dvorakova, Irina Vetter

**Affiliations:** ^1^IMB Centre for Pain Research, Institute for Molecular Bioscience, The University of Queensland St. Lucia, QLD, Australia; ^2^School of Pharmacy, The University of Queensland Woolloongabba, QLD, Australia

**Keywords:** pain, rodent, hyperalgesia, allodynia, spontaneous pain, mechanical, heat, cold

## Abstract

Rodents are commonly used to study the pathophysiological mechanisms of pain as studies in humans may be difficult to perform and ethically limited. As pain cannot be directly measured in rodents, many methods that quantify “pain-like” behaviors or nociception have been developed. These behavioral methods can be divided into stimulus-evoked or non-stimulus evoked (spontaneous) nociception, based on whether or not application of an external stimulus is used to elicit a withdrawal response. Stimulus-evoked methods, which include manual and electronic von Frey, Randall-Selitto and the Hargreaves test, were the first to be developed and continue to be in widespread use. However, concerns over the clinical translatability of stimulus-evoked nociception in recent years has led to the development and increasing implementation of non-stimulus evoked methods, such as grimace scales, burrowing, weight bearing and gait analysis. This review article provides an overview, as well as discussion of the advantages and disadvantages of the most commonly used behavioral methods of stimulus-evoked and non-stimulus-evoked nociception used in rodents.

## Introduction

Pain, as defined by the International Association for the Study of Pain (IASP), is “an unpleasant sensory and emotional experience associated with actual or potential tissue damage, or described in terms of such damage”. It is a universal human experience that in the short term serves to protect an individual from harm, but in the long term can become a debilitating condition. In humans, acute pain is defined as short-lasting (3–6 months) and is directly related to injury or tissue damage, such as a cut, burn or broken bone. The purpose of pain in the above cases is to alert an individual to withdraw from immediate tissue damaging stimuli and to prevent further damage to the site of injury during the healing process. The protective role of pain is most evident in individuals who have congenital insensitivity to pain, a rare genetic condition that results in the inability to sense tissue damaging or nociceptive stimuli (Cox et al., 2006). This normally protective response, which is absent in these individuals, leads to frequent injuries and often results in higher mortality rates early in life (Bennett and Woods, 2014). When pain continues beyond the expected time of wound healing or without a clear reason, it is termed chronic pain. Chronic pain serves no protective purpose, and depending on the severity, can be a debilitating condition that is difficult to treat, with the currently available analgesics often lacking efficacy and suffering dose-limiting adverse effects. Therefore, there is an urgent need to increase our understanding of the underlying mechanisms of pain and to develop new treatments.

Pain studies in humans are difficult to perform, are subjective, and are limited by ethical considerations, leading to the widespread use of animals as models to study pain, with the most commonly used species being mice and rats (Mogil, 2009). However, with the use of animal models come challenges relating to the appropriate quantification of behavioral responses that could be considered equivalent to pain in humans.

Despite some degree of uncertainty about the validity of the anthropomorphization of pain in animals, the capacity to experience pain and distress, particularly resulting from procedures or conditions that would cause pain and distress in humans, must be assumed unless there is evidence to the contrary. Undoubtedly, nociception, or the ability to detect a potentially harmful stimulus, is a fundamental physiological function in mammals and indeed many other species. However, as animals cannot be said to be reporting pain, any reaction to such stimuli does not necessarily evidence experience of pain (Sandkühler, 2009). It should be noted that no test can therefore measure pain in animals directly—the presumably unpleasant emotional experience of pain is inferred from pain-like behaviors which can include the withdrawal of a body part from a stimulus, reduced ambulation, agitation, an increase in grooming of the affected area, and vocalizations upon sensory stimulation. The distinction between nociception and pain thus underlines a key difference in terminology when referring to communicating and non-communicating subjects. Similarly, as it cannot be said that the animal feels pain, analgesia and analgesic intervention cannot take place—only anti-nociception and anti-nociceptive interventions can.

Accordingly, as pain cannot be directly measured in rodents, it has been necessary to develop indirect methods to quantify and evaluate pain-like behaviors in non-anesthetized animals which are reliable, reproducible, sensitive and specific (Mogil, 2009). This review article will provide an overview of the current behavioral methods that are used to assess pain behaviors in mice and rats.

### Nociception and Pain in Humans

The term nociception was coined by Charles Sherrington in the early 1900s to distinguish the sensation of pain—a result of central nervous system processing—from the physiological phenomenon of the peripheral nervous system responding to potential harmful stimuli (Dubner, 1983; Coutaux et al., 2005). Thus, the term nociception is used to describe the peripheral neuronal response to noxious stimuli, which encompasses any stimuli, being mechanical, thermal, electrical or chemical, that have the potential to damage are damaging to tissue (Dubin and Patapoutian, 2010). Typically, noxious stimuli activate nociceptors, a subset of peripheral sensory neurons, which have a range of specialized ion channels and receptors that transduce noxious stimuli into electrical signals. These neurons are pseudo-unipolar, with a peripheral branch that terminates in the skin or viscera and a central branch that terminates in the spinal cord. Nociceptive signals are then sent to the spinal cord and brain for processing as the sensation of pain. Thus, pain is an experience that encompasses both sensory and emotional components; therefore the term pain is not interchangeable with nociception.

In human patients, a distinction is made between stimulus-evoked pain and stimulus-independent or spontaneous pain. Stimulus-evoked pain is described as either hyperalgesia or allodynia, and is further subdivided on the basis of the evoked stimulus modality (e.g., mechanical, heat, cold, chemical; Woolf and Mannion, 1999). Hyperalgesia is defined as an increased or exaggerated pain response to a normally noxious stimulus, while allodynia is defined as a painful response to a normally non-noxious or innocuous stimulus. In cases of sensory loss, hypoalgesia may be present, which is defined as decreased sensitivity to a nociceptive stimulus. Stimulus-evoked pain can be evaluated in humans using quantitative sensory testing. While not in routine clinical use, quantitative sensory testing has the potential to improve patient outcomes by classifying pain based on the mechanism and choosing treatments that target that mechanism (Baron et al., 2010; Cruz-Almeida and Fillingim, 2014).

Stimulus-independent or spontaneous pain may be paroxysmal (sudden and severe) or continuous, and can be described as aching, cramping, crushing, shooting and burning (Jensen et al., 2001). Importantly, the pain appears to be spontaneous, with no identifiable stimulus. However the distinction between stimulus-evoked and non-stimulus evoked pain may be difficult to make clinically, as arguable it could be allodynia occurring from an unidentified stimulus.

#### Pain Induced by Mechanical Stimuli

Mechanical hyperalgesia and allodynia can be further subdivided into dynamic (triggered by brushing), punctate (triggered by touch) and static (triggered by pressure). Dynamic mechanical allodynia and hyperalgesia can be assessed by brushing the skin with a cotton bud, paintbrush or cotton ball, and in the case of allodynia, can be evoked by the brushing of clothing, bed sheets or towels against the skin (Jensen and Finnerup, 2014). Punctate mechanical allodynia and hyperalgesia can be evoked with a pinprick or monofilament, and in practice can be assessed by the application of von Frey filaments of varying forces (0.08–2940 mN). Static hyperalgesia can be superficial or deep and is assessed by the application of pressure to the skin or underlying tissue by a finger or using a pressure algometer (Jensen and Finnerup, 2014).

#### Pain Induced by Heat Stimuli

The exposure of peripheral sensory nerve endings to elevated temperatures can evoke sensations of warm, hot, or pain. Heat thresholds in humans can be determined by applying a metal probe to the skin that increases in temperature (starting at 32°C) until a warm-sensation threshold and heat-pain threshold is reached. Typically, the sensation of warm is elicited at temperatures of 34–37°C, while the sensation of pain is elicited at temperatures of 42–48°C (Pertovaara et al., 1996; Defrin et al., 2006; Rolke et al., 2006). These values are not absolute, as heat thresholds are influenced by the ambient temperature, rate of heating (1–10°C/s), the type (hairy or glabrous) and location of test skin, method of heat transfer, experimental design and skin temperature (for radiant heat only; Pertovaara et al., 1996; Defrin et al., 2006; Rolke et al., 2006).

#### Pain Induced by Cold Stimuli

Cold thresholds in humans can be determined in a similar manner to heat thresholds, where a metal probe is applied to skin that decreases in temperature (usually starting at 32°C) until a cooling sensation or pain threshold is reached. The sensation of pleasant or innocuous cooling is typically elicited at temperatures of ~23–29°C, while the sensation of cold pain is significantly variable, with multimodal distribution of the cold pain threshold recently reported, corresponding to modal threshold temperatures of 23.7°C, 13.2°C and 1.5°C, respectively (Lötsch et al., 2015). However, the majority of human subjects report cold pain upon cooling to at least 22°C (Defrin et al., 2002; Lötsch et al., 2015). As for heat pain, these values are likely influenced by a number of factors including ambient temperature, rate of cooling and anatomical location. Given the variability in cold pain thresholds, it may be difficult to differentiate between cold allodynia and cold hyperalgesia in the clinic.

### Nociception in Animals

#### Replacement, Reduction and Refinement

International standards and guidelines, as well as country-specific codes and legislation, have been developed to protect the welfare of animals used for research. In fact, it is a requirement for publication of *in vivo* data in high quality journals that relevant standards and guidelines are strictly adhered to (McGrath et al., 2010). The framework for these standards and guidelines are based on the principles of the 3Rs (replacement, reduction, refinement).

According to the replacement principle, the use of live animals should be replaced with *in vitro* or computational methods where possible, and if unavoidable, the use of non-sentient or less sentient animals is preferred. However, replacement or substitution of animals for nonsentient materials is difficult in pain research due to the nature of the behavioral experiments. Thus, the focus is often on reduction of the number of animals necessary to obtain data, and refinement of the method with the aim to decrease the amount of nociception caused to the animal. This can be achieved through a variety of techniques. For instance, improving data homogeneity and enhancing statistical power (Dell et al., 2002) will result in fewer animals being necessary to achieve the required confidence level (Festing and Altman, 2002). Similarly, measures to improve data quality, including appropriate randomization and blinding procedures, are key to ensure validity of the obtained results. In addition, care should be taken to design experiments that minimize distress and suffering. This includes minimizing the duration of models, replacing nocifensive model compounds for ones that cause shorter lasting nociception, or reducing the administered doses of compounds. Particular mention should also go to timely publication of data, be it positive or negative results, in order to reduce experimental duplication and unnecessary use of animals.

#### Behavioral Methods to Measure Pain-Like Behaviors

Pain cannot be directly measured in animals; instead pain is inferred from “pain-like” behaviors, such as withdrawal from a nociceptive stimulus, which is the most commonly used method to quantify nociception in animal studies. If a stimulus is applied that does not normally evoke a withdrawal response, and the animal withdraws from the stimulus, the animal is considered to have allodynia. Similarly, if a stimulus is applied that does normally evoke a withdrawal response, but the animal withdraws with an exaggerated response, the animal is considered to have hyperalgesia. However, in practice it is difficult to distinguish between allodynia and hyperalgesia in animals, and the terms allodynia and hyperalgesia are often used incorrectly or interchangeably in the literature. Similarly, the terms nociception and pain are often used interchangeably, although the term pain is rarely appropriate to use in reference to animal studies.

The outcomes of most behavioral methods used to study nociception are somewhat subjective. For example, in the case of application of a stimulus to the hind paw, the investigator must determine if the animal withdrew the hind paw due to its aversive nature, or whether the animal withdrew the hind paw for another reason (e.g., tickle, grooming, ambulation). Behaviors tend to occur on a spectrum of intensity, but are usually scored in binary as either present or absent. As each researcher cultivates a slightly different cut off point in their minds as to what constitutes a behavior on this spectrum, results can vary significantly between laboratories. Similarly, human scoring lends itself to bias, although this can be avoided with appropriate randomization, allocation concealment and blind outcome assessment (Hirst et al., 2014). It should be noted that behavioral assessment of animals in groups (even if blinded) is typically not sufficient, with a preferred method being measurements performed on animals in random order by an investigator blinded to the treatment group each animal has been allocated to.

The behavioral methods used to measure nociception in rodents can be divided into stimulus-evoked (and further subdivided by the stimulus modality—mechanical, heat, cold) and non-stimulus evoked, with the most commonly used methods discussed in this review article. For detailed protocols of these methods see Minett et al. (2011). For an overview of commonly used pain models in rodents see Gregory et al. (2013).

## Stimulus-Evoked Pain-Like Behaviors

### Mechanical Stimuli

**Figure 1:**
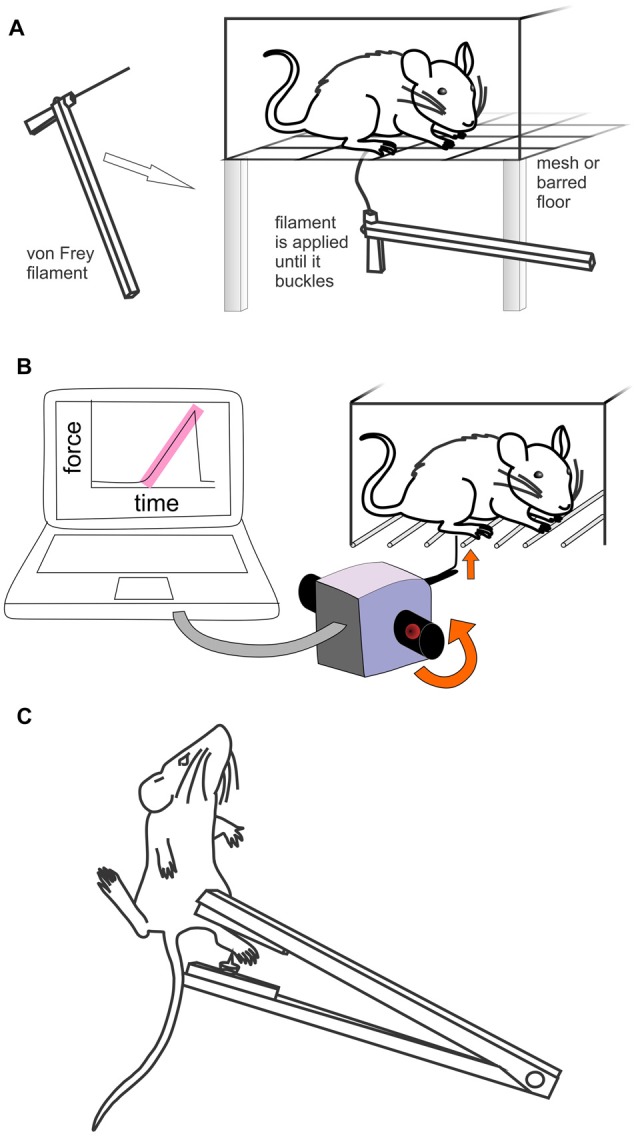
Methods used to assess mechanically evoked pain like behaviors in rodents. **(A)** Manual Von Frey. Rodents are placed individually in small cages with a mesh or barred floor. Monofilaments of differing forces are applied perpendicularly to the hind paw. If the rodent withdraws, licks or shakes the paw, it is considered to have had a positive response. **(B)** Electronic von Frey (MouseMet, TopCat Metrology). Rodents are placed individually in a small cage with a barred floor. A single, un-bending filament is applied perpendicularly to the hind paw. The force is increased by rotation of the handheld device until paw withdrawal occurs. The force ramp and paw withdrawal force are displayed by the software post-test. **(C)** Randall-Selitto test (handheld device). The rodent is restrained and the hind paw (or tail) is placed between a pointed probe tip and flat surface. The pressure is increased until withdrawal or vocalization occurs.

The presence and extent of aversive behaviors in responses to mechanical stimuli is typically determined using manual or electronic Von Frey or the Randall Selitto test, as described below (Figures 1A–C).

#### Manual Von Frey

The manual Von Frey test, developed by the physiologist Maximilian von Frey, is a method of evaluating mechanical allodynia in mice and rats. Despite the development of electronic Von Frey tests, manual Von Frey remains the gold standard for determining mechanical thresholds in mice. In this test, animals are placed individually in small cages with a mesh or otherwise penetrable bottom. A monofilament is applied perpendicularly to the plantar surface of the hind paw until it buckles, delivering a constant pre-determined force (typically 0.2–13.7 mN for mice and 5.9–98 mN for rats) for 2–5 s (Figure 1A). A response is considered positive if the animal exhibits any nocifensive behaviors, including brisk paw withdrawal, licking, or shaking of the paw, either during application of the stimulus or immediately after the filament is removed. While the plantar surface of the hind paw is the most commonly used area for testing, other areas of the body, including the dorsal surface of the hind paw or the abdomen can also be used (Minett et al., 2014). Different methodological approaches are used to determine mechanical sensitivity using manual Von Frey, including the “up-down”, “ascending stimulus” or “percent response” method, all of which will be discussed below.

**Figure 2:**
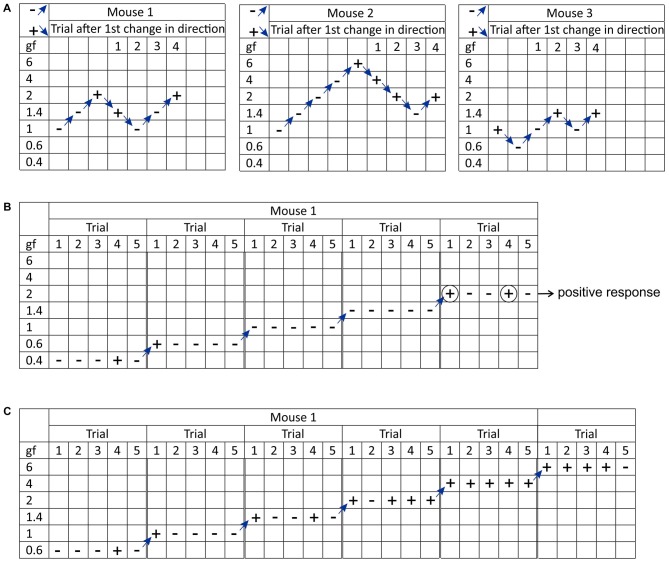
Visual representation of the different methodological approaches used to determine mechanical sensitivity using manual Von Frey. **(A)** The “up-down” method. The test begins by assessing the response to a filament estimated to be close to the 50% withdrawal threshold (in this case 1 grams-force). If there is no response, the next filament with a higher force is tested; if there is a response, the next lower force filament is tested. This continues until at least four readings are obtained after the first change of direction. The sequence of outcomes ( − for no response or + for response) is recorded and later used to calculate the 50% withdrawal threshold. **(B)** The “ascending stimulus” method. The test begins by assessing the response to a filament of the lowest force (in this case 0.4 grams-force) for a set number of applications (in this case five times). If the response rate is less than 40% (i.e., a withdrawal response is elicited in none or one out of five applications) the next filament is tested. If the response rate is 40% or more (i.e., withdrawal response is elicited in two or more out of five applications) testing stops and the force of the last von Frey filament is designated as the mechanical withdrawal threshold (in this case 2 grams-force). **(C)** The “percent response” method. In this test, monofilaments of varying forces (in this case 0.6, 1, 1.4, 2, 4 and 6 grams-force) are applied in ascending order an equal number of times (in this case five times) and the response to each trial is recorded.

The “up-down” Von Frey method is used to determine the mechanical force required to elicit a paw withdrawal response in 50% of animals, based on the statistical formula used to determine LD_50_s (Dixon, 1980; Chaplan et al., 1994). The experiment begins by testing the response to a filament estimated to be close to the 50% withdrawal threshold. If there is no response, the next filament with a higher force is tested; if there is a response, the next lower force filament is tested. This continues until at least four readings are obtained after the first change of direction, and the sequence of outcomes (− for no response or + for response) is recorded (Figure 2A). At least six responses around the estimated threshold are required for optimal calculation of the 50% threshold (Dixon, 1980). The 50% threshold is then calculated using the formula: 50% threshold (*g*) = 10^(X+kd)^/10^4^, where *X* = the value (in log units) of the final von Frey filament, *k* = tabular value for the response pattern (see Appendix 1 in Chaplan et al., 1994) and *d* = the average increment (in log units) between von Frey filaments. The “up-down” method is practically limited by commercial filaments that are not equally spaced (therefore, the average increment is used for *d*), incorrect labeling of the force in log units, and the need for the first filament to be close to the mean threshold (which may be unknown; Bradman et al., 2015). A disadvantage of this method is that the number of measurements per animal is variable and that it requires repeated, time-intensive measurements, which may lead to sensitization or learnt responses.

The “ascending stimulus” method provides an estimate of the mechanical withdrawal threshold. It is an estimate, as the force applied by manual von Frey filaments can only be applied in discrete steps, and not continuously like electronic von Frey (see below). The method is based on the application of monofilaments with increasing force until a withdrawal response is elicited, and the force of the von Frey filament that elicits this positive response is designated as the mechanical withdrawal threshold (Figure 2B). The criterion that constitutes a positive response to a filament varies between laboratories, with 20%–40% withdrawal response rates over 5–10 applications being used typically (Scholz et al., 2005; Minett et al., 2011). An advantage of this method is that it avoids excessive application of Von Frey filaments that elicit aversive behaviors.

In the “percent response” method, several Von Frey filaments of varying forces are applied in ascending order an equal number of times (usually 5–10 applications) regardless of response, and the number of positive responses to each filament is converted to a percent response (Kim and Chung, 1992; Chaplan et al., 1994; Figure 2C). The advantage of this approach is that each animal receives the same number and type of stimuli, although the number of tests per hind paw could exceed 50 (e.g., five different von Frey filaments each applied 10 times), which is not only time consuming, but potentially exposes animals with mechanical hind paw sensitivity to a disproportionate number of more aversive stimuli.

The manual Von Frey tests enable quantification of mechanical thresholds in unrestrained animals, which removes the risk of handling-induced stress. However, this also requires animals to be acclimatized to the cages, to ensure ambulation and exploratory behaviors, which could be misinterpreted as a positive response, are kept to a minimum. While rats tend the habituate quickly (<15 min), mice can take up to an hour or more to settle in the cage before testing can begin, which can be time consuming (Chaplan et al., 1994; Minett et al., 2011). Testing should also be avoided while the animal is engaged in grooming behaviors, as this can produce false negative responses. Consistent and precise placement of the filament is important to reduce intra-subject and inter-subject variability, with the specific placement dependent on the innervation territories of the test area and the model used. For example, in the spared nerve injury model, the tibial and common peroneal nerves are axotomized, leaving only the sural nerve intact. In this model, the lateral plantar skin of the hind paw, which is the area of innervation of the sural nerve, has the greatest reduction in mechanical thresholds compared to other innervation areas of the plantar skin (Decosterd and Woolf, 2000).

Rodents may also respond to initial contact with a filament with a “touch-on” response. A touch-on reaction is more likely to occur if the filament is not applied perpendicularly, if the filament is not applied smoothly, or if the filament moves horizontally during application, inducing scratching. In addition, rodents are intelligent and can learn that premature withdrawal will result in less human interaction and stimulation. Experimenters experienced in the technique are able to distinguish between “touch on” or false positive responses, however, this can be difficult for inexperienced researchers and extensive training is usually required to produce high quality data.

#### Electronic Von Frey

Electronic Von Frey systems operate under similar principles as manual von Frey, except that a single, un-bending filament is applied with increasing force until a paw withdrawal response is elicited. The force at which this response occurs is recorded automatically by the apparatus and is designated as the paw withdrawal threshold. The main advantage of electronic Von Frey compared to manual Von Frey is that an increasing force is applied by a single filament. This therefore provides measure of paw withdrawal threshold on a continual scale, as the force is applied continuously and not in steps. In addition, the experimental time is dramatically reduced, as few applications (usually 3–4) are needed to determine the paw withdrawal threshold (Deuis et al., 2014, 2015). Despite these advantages of automation, experimenters still need to be experienced to distinguish true responses from “touch-on” responses and ambulation. In addition, as for manual Von Frey tests, animals still need to be habituated to the cages until exploratory behaviors have ceased. While several systems are commercially available, the Dynamic Plantar Aesthesiometer (Ugo Basile) and MouseMet or RatMet (TopCat Metrology) are particularly robust and user-friendly systems.

The Dynamic Plantar Aesthesiometer (or Plantar Von Frey), houses rodents in an enclosure with a mesh screen floor, under which a movable touch-stimulator unit is placed. Under the direction of the researcher, the apparatus applies a von Frey (0.5 mm) filament to the plantar surface, increasing the force incrementally (0–50 g) until the paw withdrawal threshold is reached. The device automatically records the force at which paw withdrawal occurs and the rate at which the force is applied can be changed. In addition, a programmable “hold” step with constant force application can also be incorporated in the experimental setup to determine the time to withdrawal (Lu and Schmidtko, 2013).

The MouseMet or RatMet electronic Von Frey systems deliver a mechanical stimulus via a hand-held probe (Figure 1B). In contrast to other Von Frey setups, animals are housed in individual enclosures with bars, rather than mesh, to help maximize the surface area of the hind paw available for application of the filament. However, as the testing surface can influence the results of von Frey, it is possible that values obtained using MouseMet or RatMet are not directly comparable to other methods (Pitcher et al., 1999). The RatMet or MouseMet von Frey filament (0.3 and 0.5 mm tip diameter, delivering forces of 1–80 g and 0.1–7 g, respectively) is placed against the plantar surface of the paw and the force is linearly increased via rotation of the device handle (Deuis and Vetter, 2016). In addition to displaying force at which paw withdrawal occurs, the rate at which the force was applied is also displayed post-test by the software, to ensure the force ramp was applied consistently. A soft transducer ensures minimal vibration and reduces “touch-on” responses. Both instruments have been validated against manual von Frey filaments and found to produce less variable data in addition to being easier to use.

It should be noted that the absolute values obtained using manual and electronic Von Frey can differ significantly. For example, in C57BL/6 mice, the 50% withdrawal threshold determined using manual Von Frey is ~0.6–1 g, while using electronic Von Frey, the paw withdrawal thresholds are generally higher, with values of ~4 g obtained using MouseMet and ~6 g using Dynamic Plantar Aesthesiometer (Petrus et al., 2007; Minett et al., 2014; Deuis and Vetter, 2016; Gritsch et al., 2016). While the underlying protocols and principles are different, it remains to be determined if electronic Von Frey activates a different subset of sensory neurons compared to manual Von Frey (e.g., high-threshold vs. low threshold mechanoreceptors). However, irrespective of the method used, the endpoint is paw withdrawal to a stimulus that is not normally aversive, and thus both methods can measure mechanical allodynia.

#### Randall-Selitto Test

The Randall-Selitto or paw pressure test was developed as a tool to assess response thresholds to mechanical pressure stimulation and is often considered a measure of mechanical hyperalgesia (Figure 1C; Randall and Selitto, 1957). This test involved application of an increasing mechanical force to the surface of the paw or tail until withdrawal or vocalization occurs. In practice, this test is useful for assessment of nociceptive thresholds in rats rather than mice as animals need to be heavily physically restrained with the tested paw held out, and mice rarely tolerate such handling (Anseloni et al., 2003; Minett et al., 2011; Santos-Nogueira et al., 2012). The exception is use of the test on the tail of mice (Minett et al., 2014), although this may not be useful to assess nociceptive behaviors in commonly used models that are localized to the hind paw.

The Randall-Selitto test can be performed using bench-top (e.g., Analgesy-Meter, Ugo Basile; Paw and Tail Pressure Meter; Harvard Apparatus) or hand-held devices (e.g., Paw Pressure Test Apparatus, IITC) with animals either restrained in a hammock that provides access to the hind paws, a towel, or in a plastic cone or cylinder. To obtain reliable data, animals need to be habituated to the restraint method and experimental apparatus, which can become very time-intensive. Mechanical pressure is applied focally to the dorsal or plantar surface of the hind paw or tail, which is placed between a pointed probe tip and a flat surface. The pressure is then increased at a constant rate until a nociceptive behavioral response is observed.

The withdrawal response is detected visually by the researcher, resulting in subjective measurement of the threshold. It should be noted that the paw withdrawal threshold can be considered a measure of spinal reflex, with some researchers favoring vocalization as an end-point (Winter and Flataker, 1965; Kayser and Christensen, 2000; Santos-Nogueira et al., 2012). These measures can have a profound effect on the apparent anti-nociceptive efficacy of test compounds and should thus be carefully considered during experimental design (Winter and Flataker, 1965). However, rodents do not vocalize in the audible range unless the pain is severe, making use vocalization as an endpoint ethically limited (Mogil, 2009). The use of ultrasonic (inaudible) vocalization as an endpoint has also been studied, however it has not consistently been shown to increase in response to noxious stimuli (Han et al., 2005; Wallace et al., 2005; Williams et al., 2008). While the Randall-Selitto test often results in similar types of outcomes to the Von Frey filament tests (Santos-Nogueira et al., 2012), the mechanical stimulation differs fundamentally from Von Frey filaments, which may also activate low-threshold mechanoreceptors in addition to nociceptors.

### Heat Stimuli

#### Tail Flick Test

**Figure 3:**
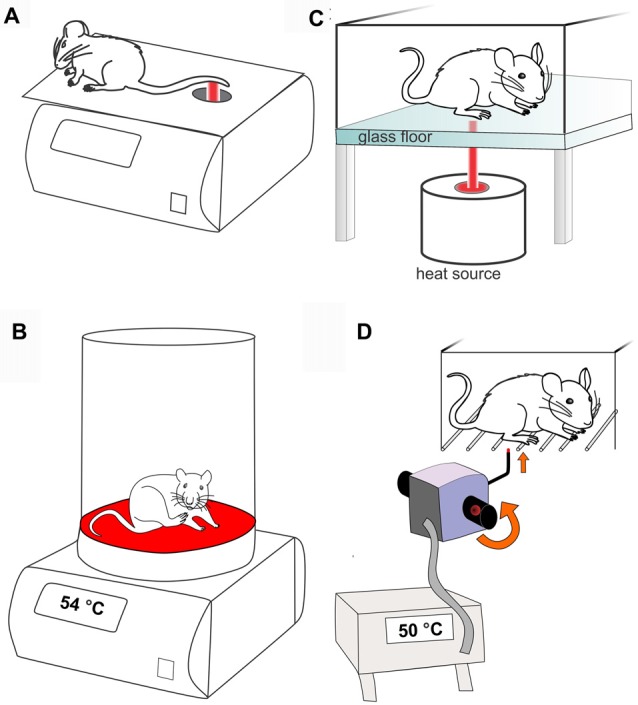
Methods used to assess heat-evoked pain like behaviors in rodents. **(A)** Tail flick test (radiant heat). Rodents are restrained and a focused beam of light is applied to tail. The time taken to “flick” or withdraw the tail from the heat stimulus is recorded. **(B)** Hot plate test. In the conventional hot plate test the rodent is placed on a metal surface maintained at a constant temperature (in this case 54°C) and the time taken to elicit a nocifensive behavior (e.g., hind paw withdrawal or licking) is recorded. **(C)** Hargreaves test. Rodents are placed individually in small enclosures with a glass floor. A radiant or infrared heat source is focused on the plantar surface of the hind paw and the time taken to withdraw from the heat stimulus is recorded. **(D)** Thermal probe test. Mice are placed individually in small cages with a barred floor. A small metal probe is applied to the hind paw, and heating is triggered by rotation of the handheld device until the mouse withdraws the paw. The device automatically records the temperature that paw withdrawal occurred (in this case 50°C).

The tail flick test, first described in 1941, involves application of a heat stimulus to the tail of mice and rats, and the time taken for the tail to “flick” or twitch is recorded (D’Amour and Smith, 1941; Figure 3A). The heat stimulus applied can be radiant heat, where a focused beam of light is applied to the tail, or hot water, where the distal end of the tail is immersed into a water bath set at a constant temperature between 46°C and 52°C, with the latter requiring no specialized equipment. Both versions of the test require the animal to be loosely restrained.

While relatively quick and easy to perform, an important consideration with the tail flick test is that a similar behavioral response can be observed in spinally transected rats, consistent with the notion that the tail withdrawal response is a spinal reflex, rather than an indication of pain behaviors involving higher brain centers (Irwin et al., 1951). This suggests that the tail flick response may be impacted by changes in motor processing (Chapman et al., 1985). However, the contribution of supraspinal processing to the tail flick response depends at least in part on the heating slope and temperature, with stimuli that lead to more delayed withdrawal responses generally considered to involve higher central nervous system functions considered necessary to process “pain” (Jensen and Yaksh, 1986). In addition, skin and ambient temperature, the location of stimulus application on the tail, as well as learnt avoidance behaviors can affect the withdrawal response (Yoburn et al., 1984; Berge et al., 1988). The clinically translatability of the tail flick test is therefore unclear. While the method carries the disadvantage that the rodent has to be restrained, the tail flick test is of very short duration so handling can be minimized easily.

#### Hot Plate Test

The hot plate test, first described in 1944, can be used to determine heat thresholds in mice and rats (Woolfe and Macdonald, 1944). Unlike the tail flick test, the hot plate test and other tests that apply heat stimuli to the hind paws are considered to integrate supraspinal pathways, as rats with spinal transection do not withdraw the hind limbs in the hot plate test (Giglio et al., 2006). In the conventional hot plate test, an unrestrained mouse or rat is placed on a metal surface maintained at a constant temperature, usually between 50°C and 55°C, and the response latency, which is the time taken to observe a nocifensive behavior, is recorded by the investigator (Figure 3B). Nocifensive behaviors include forepaw withdrawal or licking, hind paw withdrawal or licking, stamping, leaning posture and jumping (Espejo and Mir, 1993). While forepaw withdrawal often occurs first, hind paw withdrawal or licking is considered to be a more reliable indicator of nociception, as the forepaws are frequently used in grooming and exploration and are not consistently in contact with the metal surface (Woolfe and Macdonald, 1944; Minett et al., 2011). If no nocifensive behaviors are observed, the animal must be removed from the hot plate after pre-determined cut-off time to prevent tissue damage. Alternatively, the number of flinches over a set period of time can be recorded at a specific temperature (Yalcin et al., 2009; Deuis et al., 2013; Zimmermann et al., 2013), although care must be taken that the chosen temperature and duration do not induce tissue damage or nocifensive behavior in naïve animals.

The dynamic hot plate test, first described in 1984, uses an increasing temperature ramp rather than a constant temperature. In this test, the unrestrained mouse or rat is placed on a metal surface starting at a non-noxious temperature (<42°C), and the temperature is increased at a constant rate until a nocifensive behavior is observed. The temperature at which this occurs is designated as the response temperature (Ogren and Berge, 1984; Tjolsen et al., 1991). The response temperature is dependent on the starting temperature, ambient temperature and rate of heating, with faster heat ramps resulting in higher response temperatures (Tjolsen et al., 1991; Yalcin et al., 2009). As for the static hot plate test, cut off times should be carefully designed and strictly adhered to in order to avoid unnecessary nociceptive stimulation and tissue damage.

Depending on the species and strain of rodent used, at least 12 different behaviors have been noted in the hot plate test, including sniffing, grooming, stamping of the legs, freezing, licking, leaning and jumping (Espejo and Mir, 1993). Some of these behaviors can be sensitive to analgesics, although differences are observed depending on the type of behavior quantified. For example, paw licking is diminished by opioids but not other analgesics, while other behaviors can also be affected by other classes of analgesics (Ankier, 1974; Hunskaar et al., 1985). Data quality is usually improved if the time to occurrence of any behavior, rather than specific behavior types, is recorded, and if lower temperatures are used (Carter, 1991; Plone et al., 1996). It is plausible that differences in behavior may relate to the type of sensory fiber activated. In anesthetized rats, steep temperature gradients and high skin temperatures are associated with activation of Aδ fibers, while slower heating and lower temperatures lead to firing of C fibers (Yeomans and Proudfit, 1994, 1996; Yeomans et al., 1996).

An additional confounding factor in the hot plate test is the tendency for learned behavioral responses, which lead to diminished reaction times during subsequent exposures to the hot plate (Gamble and Milne, 1989; Plone et al., 1996). Thus, the hot plate test can produce greatly variable data, even within laboratories. An additional disadvantage of the hot plate test is that all four paws and the tail are exposed to the heat stimulus. While this is generally not an issue when testing the anti-nociceptive effects of compounds delivered systematically or when phenotyping transgenic mice, this may confound the results for unilateral models of pain or for compounds administered by intraplantar injection. This problem can be overcome by restraining the rodent and only placing the plantar surface of a one hind paw on the metal surface and recording the time to withdrawal, however this method requires significant handling and associated stress (Menéndez et al., 2002).

#### Hargreaves Test

The Hargreaves test, first described in 1988, is a method used to quantify heat thresholds in the hind paws of mice and rats upon application of a radiant or infrared heat stimulus (Hargreaves et al., 1988). The Hargreaves test is usually carried out using a glass bottom enclosure, which can be heated to minimize errors arising from heat sink effects. A radiant or infrared heat source is positioned underneath the animal and aimed at the plantar surface of the hind paw (Figure 3C). The time taken to withdraw from the heat stimulus is recorded as the withdrawal latency, and depending on the model of the Hargreaves apparatus, may either be recorded manually by the investigator or automatically by the apparatus. The intensity of the light source should be adjusted to produce withdrawal latencies of 10–12 s in naïve animals, providing a sufficient window to detect heat allodynia and hypoalgesia, with a pre-determined cut off time to prevent tissue damage. The Hargreaves test permits measurement of ipsilateral and contralateral heat thresholds, allowing each animal to serve as its own internal control in unilateral pain models. In addition, the Hargreaves test enables quantification of heat thresholds in unrestrained animals, reducing the likelihood of stress-induced responses. However, this requires the animals to be acclimatized to the apparatus to minimize ambulation so that withdrawal latencies can be accurately determined. While generally not an issue in rats, which are reported to only require 5 min of habituation (Hargreaves et al., 1988), the habituation time in mice is often reported to be 30 min or longer (Harvey and Dickenson, 2009; Guilford et al., 2011; O’Brien et al., 2013), precluding testing of compounds with short duration of action or pain models of limited duration.

A disadvantage of the Hargreaves test is that the paw withdrawal time is recorded rather than directly measuring the paw withdrawal temperature. While paw withdrawal temperature can be derived from the time to withdrawal (Hargreaves et al., 1988), the actual temperature applied to the skin would need to be experimentally determined by attaching a thermocouple probe to the skin. To address this, a modified Hargreaves test has been reported, that utilizes a feedback-controlled radiant heat source to apply a constant temperature to the hind paw. Increasing temperatures are applied for 10 s from 35°C to 70°C in intervals of 2.5°C until a paw withdrawal behavior is observed (Banik and Kabadi, 2013). While validated using an incisional model of pain in rats, this method takes longer to perform than the normal Hargreaves test and is not available to purchase commercially.

#### Thermal Probe Test

The thermal probe test (MouseMet Thermal, Topcat Metrology) is a novel method recently described to quantify heat thresholds in mice (Deuis and Vetter, 2016). This test can be carried out using the same mouse enclosures as the electronic von Frey test (MouseMet) and is based on the application of a 2 mm thermal probe to the hind paw. Rotation of the handle of the hand-held device initiates heating from room temperature to 60°C at a rate of 2.5°C/s, which is terminated automatically by paw withdrawal, removal of the probe from the paw by the operator, or on reaching a predetermined cut-out (usually 60°C; Figure 3D). The temperature at which paw withdrawal occurred is automatically recorded, enabling recording of the paw withdrawal temperature without the delay of an investigator manually noting the temperature (Deuis and Vetter, 2016).

Similar to the Hargreaves test, the thermal probe tests enables quantification of ipsilateral and contralateral heat thresholds in unrestrained mice, but with a shorter habituation time of 5–10 min. In naïve C57BL/6 mice, the paw withdrawal temperature occurs at ~50°C, and in unilateral models of inflammation the paw withdrawal temperature reduces to 43–44°C, providing a sufficient window to detect heat allodynia as well as hypoalgesia (Deuis and Vetter, 2016; Deuis et al., 2017a). The main advantage of the thermal probe test is that the mice are placed in individual runs standing on bars instead of glass enabling access to the plantar surface, which allows simultaneous assessment of mechanical thresholds by von Frey, removing the need for acclimation in two different enclosures. While application of a contact heat stimulus achieves consistent and efficient thermal transfer, it also represents a mechanical stimulus that may lead to premature paw withdrawal in models with mechanical allodynia. However, the force required to trigger probe heating is low (~1 g), adjustable, and the surface of the heating probe is rather large compared to the punctate von Frey filaments. Accordingly, the development of thermal allodynia was demonstrated to be independent to the development of mechanical allodynia in unilateral models of carrageen-induced inflammation and burn injury (Deuis and Vetter, 2016). Nonetheless, the thermal probe test remains to be validated in other pain models that cause more pronounced mechanical allodynia.

A major advantage of the thermal probe test is the reduced time required for acclimatization to the testing environment, enabling characterization of models or compounds with short duration of action, as well as testing of mechanical and thermal thresholds in the same enclosure. In addition, welfare benefits in form of testing of unrestrained mice and exposure of only a single hind paw to a noxious heat stimulus are favorable. A modified version of the thermal probe test suitable for quantifying heat thresholds in rats is still to be developed.

### Cold Stimuli

#### Cold Plate Test

The cold plate test is one of the simplest assays to determine behavioral responses to both noxious and innocuous cold temperatures in both mice and rats. A number of endpoints can be obtained from the cold plate test, similar to the hot plate test. First, the response to a specific temperature (typically −5°C to 15°C) can be recorded (Allchorne et al., 2005). Here, the rodent is placed on the plate after it has been cooled to the desired temperature and the time taken to evoke nociceptive behavior such as shaking, jumping or licking in the animal is recorded as the response time. Second, the number of flinches over a set period of time can be recorded at a specific temperature (Deuis et al., 2013; Zimmermann et al., 2013). Third, aversive response to a cooling ramp can be used to determine the cold response threshold (Yalcin et al., 2009). It should be noted that rather than flinching or licking, some rat strains tend to simply avoid weight bearing on the affected paw or reposition their stance to minimize contact with the cool surface, so all observation should be adapted to the specific model animal.

These techniques provide an insight into how sensitive to cold temperatures the animal is, and thus provides an indirect measure of cold-induced hyperalgesia and allodynia. Advantages of the cold plate test are its relative speed and the ability for accurate temperature control. Unlike the hot plate test, the cold plate test is particularly useful for models where only one paw is affected or sensitized by the experimental compound (unilateral pain) as guarding of the affected limb can be easily achieved, and thus can be easily quantified. In contrast, quantification of aversive behaviors to cold can be more difficult in models with bilateral cold sensitivity, as paw lifting/guarding is less easy to discern and behavioral signs such as jumping or vocalizations occur rarely, although this depends at least in part on the strain of animal used. Quantification of more subtle behaviors, such as walking backwards or grooming of the front paws has thus been proposed as alternatives to quantify cold pain behaviors, although the validity of this approach has not been systematically assessed.

#### Acetone Evaporation Test

**Figure 4:**
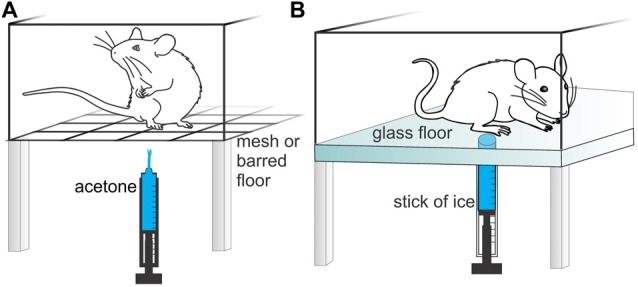
Methods used to assess cold-evoked pain like behaviors and temperature preference in rodents. **(A)** Acetone evaporation test. Rodents are placed individually in small cages with a mesh or barred floor. Acetone is applied to the hind paw and the nocifensive response(s) is counted, timed or scored. **(B)** Cold plantar assay. Rodents are placed individually in small enclosures with a glass floor. A cold stimulus is applied to the hind paw using a cut off syringe filled with dry ice or wet ice and the time to paw withdrawal is recorded.

The acetone evaporation test, first described in 1994, is a technique used to measure aversive behaviors triggered by evaporative cooling and is typically considered as a measure of cold allodynia (Carlton et al., 1994; Choi et al., 1994; Vissers and Meert, 2005). The test is carried out on mesh floor and acetone is dabbed or sprayed on the plantar surface of the hind paw (Figure 4A), eliciting cooling of the skin to innocuous temperatures of 15–21°C (Colburn et al., 2007; Leith et al., 2010), although the actual temperature varies with ambient temperature, skin temperature, and the amount of acetone applied. Exposure of the hind paw to acetone does not evoke paw withdrawal in lightly anesthetized animals (unlike the tail flick assay), while ethyl chloride application achieves skin temperatures approaching 5°C or less, and is generally considered a noxious cold stimulus (Leith et al., 2010). Consistent application of acetone can be challenging, as acetone has a lower surface tension than water (25.2 mN/m and 72.8 mN/m respectively), making it difficult to form uniform drops with a pipette or syringe (Vazquez et al., 1995), with some laboratories opting to use a spray instead (Yamamoto et al., 2016).

Sensitivity to cold is recorded either by quantifying the number or duration of nocifensive responses, or scoring of the severity of the response (e.g., 0, no response; 1, brisk withdrawal or flick of the paw; 2, repeated flicking of the paw; 3, repeated flicking of the hind paw and licking of the paw; Colburn et al., 2007; Xing et al., 2007). Water heated to 30°C and applied the same way is usually used as a control (Carlton et al., 1994; Choi et al., 1994). As the nocifensive response can be too fast for an investigator to quantify in real time, video recordings that are played back in slow motion may be required to accurately analyze the response to acetone. Despite being considered an innocuous stimulus, naïve mice and rats can have a nocifensive response to the application of acetone, likely due to the concurrent olfactory or auditory (spray) stimuli, reducing the sensitivity of the assay (Colburn et al., 2007; Yamamoto et al., 2016). Nevertheless, the acetone evaporation test has been validated in multiple models of inflammatory and neuropathic pain in both mice and rats (Choi et al., 1994; Colburn et al., 2007; Yamamoto et al., 2016). An additional advantage of the acetone drop test is the unilateral application of the thermal stimulus, enabling comparison to the contralateral side in unilateral models, and a perhaps more ethical stimulus in bilateral models.

#### Cold Plantar Assay

In the cold plantar assay, the animal is placed in an enclosure with a clean glass floor. A cold stimulus is delivered by applying a cut off syringe filled with dry ice (for temperature ranges of 5–12°C) or wet ice (temperature of 17°C) to the glass underneath the paw (Brenner et al., 2012, 2015; Figure 4B). Cooling of the glass leads to unilateral exposure of the hind paws to a cooled surface, the temperature of which can be determined by attaching a thermocouple probe to the glass or skin. The latency to paw withdrawal is recorded and used to quantify cold allodynia and hyperalgesia. However, while application of the ice pellet to the glass generates a cooling ramp that can be approximately correlated to estimates of the paw withdrawal temperature, the paw being tested needs to remain in contact with the glass to achieve efficient temperature transfer. Nonetheless, consistent measurements are possible in acclimatized animals, although guarding or altered weight distribution may lead to errors.

### Temperature Preference Test

**Figure 5:**
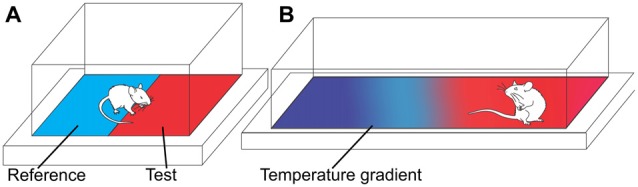
Temperature preference assays. **(A)** Two-temperature choice assay. Rodents are allowed to freely move between a reference plate (neutral temperature) and test plate. The time spent on the test plate relative to the reference plate is measured over a set period of time. **(B)** Continuous temperature gradient assay. Rodents are allowed to freely move along a liner or circular surface with a temperature gradient. The time taken to settle in a temperature zone and/or the temperature of the chosen zone is recorded.

The temperature preference is used as a surrogate measure of thermal aversion and aims to assess temperature preference in rodents. In its simplest form, the animal can choose between two adjacent areas maintained at different temperatures. This test is also referred to as the two-temperature choice assay or thermal place preference test and can be used to assess both cold or heat avoidance or preference (Moqrich et al., 2005). Typically, the experimental setup consists of a test plate at a fixed temperature (usually between 5°C and 55°C) that is placed adjacent to a reference plate at neutral temperature (usually between 25°C and 30°C; Figure 5A). To quantify temperature sensitivity, the time the animal spends on the test plate relative to the reference plate is measured over a set period and is then compared to control animals.

Alternatively, a continuous temperature gradient—either in linear (Figure 5B) or circular form—can be used to determine the preferred temperature in freely moving animals (Moqrich et al., 2005; Touska et al., 2016). While the underlying principle of the temperature gradient assay is similar to the two-temperature choice assay, the animal is free to explore along the gradient (usually between −4°C and 65°C over a length of 120 cm) until they settle within their preferred temperature or comfort zone. To evaluate thermal sensitivity, the time taken to settle in a temperature zone, as well as the temperature of the chosen zone, can be compared to control animals.

Temperature preference assays are typically relatively fast and require little rodent handling or restraint (Morgan et al., 2012). However, as the animal is required to explore, habituation, time of day, and light levels may significantly affect results and diminish reproducibility (Millecamps et al., 2005; Balayssac et al., 2014). The biggest challenge of temperature preference tests is choosing the optimal temperature pairs, so that preference for one side is either exaggerated or overcome in experimental animals compared with control animals. For example, in a rat model of carrageenan-induced inflammation, significantly altered plate preference was only observed for temperatures of 15°C and 45°C relative to the test plate maintained at 25°C (Balayssac et al., 2014). While such behavioral changes may correlate to thermal allodynia or hyperalgesia, the contribution of additional sensations or complex behaviors to the preferred environmental temperature cannot be ruled out. Nevertheless, the temperature preference test has been used extensively in the study of the role of thermosensitive transient receptor potential (TRP) channels in thermal nociception including TRPA1, TRPM8, TRPM3, TRPV3 and TRPV4 (Bautista et al., 2007; Knowlton et al., 2010; Huang et al., 2011; Vriens et al., 2011; Touska et al., 2016).

## Non-Stimulus Evoked Nociception

In humans, spontaneous or background pain is pain that occurs without an identifiable stimulus. Spontaneous pain can be quantified in humans by asking them to describe their pain using a numeric pain scale (0–10), visual analog scale (transected line) or verbal scale (no pain to worst pain; Gaston-Johansson et al., 1990; Wibbenmeyer et al., 2011). Obviously this cannot be done in rodents, making spontaneous pain difficult to quantify; however new methods to evaluate spontaneous pain are increasingly being reported, including grimace scales, burrowing assays, gait analysis, weight bearing and automated behavioral analysis (for a summary on behavioral tests used in non-stimulus evoked nociception, see Tappe-Theodor and Kuner, 2014). As many animal models of pain using stimulus-evoked measures of nociception have failed in the past to translate into the clinic, spontaneous pain as an efficacy endpoint may be more relevant to the human condition and increase the clinical validity of animal models of pain in the future (Mogil, 2009).

### Grimace Scales

**Figure 6:**
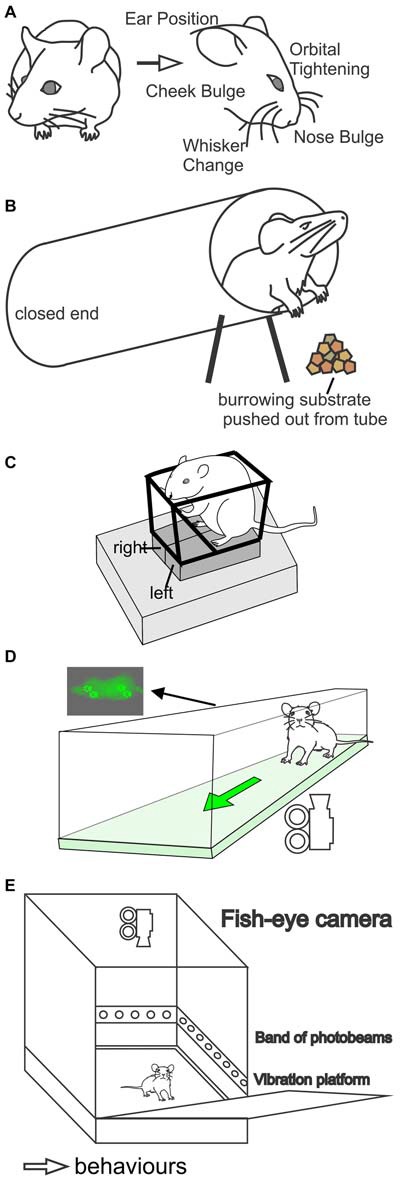
Methods used to assess non-stimulus evoked pain behaviors in rodents. **(A)** Grimace scales. Facial expression is subjectively scored for severity of pain based on five facial features (ear position, eye closing, cheek bulging, whisker position, and nose bulging). **(B)** Burrowing assay. A burrow is placed in the cage of a rodent filled with a suitable substrate (such as food pellets, sand, or marbles). The amount of substrate displaced over a set period of time is recorded. Pain in rodents is associated with decreased burrowing behaviors. **(C)** Weight bearing (incapacitance test). The rodent is placed in an inclined holder with the hind paws resting on two separate pressure sensors. Weight distribution between the hind paws is recorded. **(D)** Gait analysis (Catwalk XT, Noldus). In this assay rodents walk freely across an enclosed elevated glass floor. A camera below records the paw prints, which are illuminated by internally reflected light in the glass. A number of parameters are automatically analyzed by the software, including paw intensity, print area, stance phase duration (time spent on paw) and swing phase duration (time spent off paw). **(E)** Behavioral Spectrometer (Behavioral Instruments). Rodents are placed in an enclosed box with a camera, accelerometer and wall-mounted photobeams for a set period of time. The software records the duration of different behavior types, including movement, grooming and rearing behaviors.

Facial expressions of mice can be used to score the subjective intensity of pain. In the Mouse Grimace Scale (Figure 6A), five facial features are scored: orbital tightening, nose bulge, cheek bulge, ear position, and whisker position (Langford et al., 2010). Orbital tightening is the narrowing of the orbital area and tightly closing or squeezing of eyes. Nose bulge refers to the bulge noticeable on the bridge of the nose, whereas cheek bulge refers to the rounded projection of the cheek muscle compared to its typical appearance. Ear position denotes the ears being pulled back and apart from their standard position (may feature vertical ridges). Finally, whisker change refers to change in whisker position (may be backward, forward, or clumped together). The severity of these expressions varies with the severity of perceived “pain”, and is graded on a scale with 0 being normal, 1 being moderately, and 2 being severely changed features. The Mouse Grimace Scale is highly accurate, but generally requires significant amounts of nociception in order to elicit a visible response, which limits its use. In addition, while a “pain face” is apparent in some models of moderate duration (including acetic-acid induced writhing, the second phase of the formalin test, post-surgical pain and after intraplantar injection of mustard oil or zymosan) short nociceptive stimuli (including tail clip and tail-flick tests), and models of long-lasting neuropathic pain (including the chronic constriction injury and spared nerve injury model), are not associated with altered facial features (Langford et al., 2010).

A similar scale has also been developed for rats. The Rat Grimace Scale is also scored 0–2 depending on observed changes in facial features, and evaluates the extent of orbital tightening, nose/cheek flattening, ear change and whisker change (Sotocinal et al., 2011). The Rat Grimace Scale is able to detect spontaneous pain induced by intraarticular kaolin-carrageenan, intraplantar Complete Freund’s Adjuvant (CFA), and post-surgical pain. Both the mouse and rat grimace scale are limited by the need for extensive training of the observer and a degree of subjectivity, which could lead to variability. Development of the Rodent Face Finder^®^, which captures stills of rodent faces that a researcher then scores according to the relevant scale, has automated some of the experimental process (Sotocinal et al., 2011) albeit development of automatic facial expression scoring will make this approach even more widely useful. In line with this, the uptake of grimace scales to study pain in the scientific community has been low, with approximately 30 studies published using the method in rodents since its introduction by Langford et al. (2010). However, it is proving useful as a tool to monitor animal welfare, not only in rodents, but also in other species, with grimace scales being developed for pigs, sheep and horses (Matsumiya et al., 2012; Miller and Leach, 2015; Dalla Costa et al., 2016; Hager et al., 2017; Viscardi et al., 2017). Therefore grimace scales have the potential to monitor and improve the welfare of animals used not only in research, but also in farming and industry.

### Burrowing

Burrowing, a spontaneous and self-motivated behavior, can be used as a measure of spontaneous or non-stimulus evoked nociception in mice and rats. A burrow filled with a suitable substrate (such as food pellets, sand, or marbles) is made from a long tube sealed at one end, secured and lifted by screws on the other end to prevent non-burrowing behaviors from displacing the substrate inside (Figure 6B). The burrows are placed in the rodent’s cage for a pre-determined duration and the amount of material displaced is weighed and recorded (Deacon, 2006b; Jirkof et al., 2010). Allowing rodents to have several trial runs prior to the actual experiment can increase burrowing behaviors and reduce variability (Deacon, 2006b). When rodents are unwell, the amount of material removed from the burrow is decreased. An advantage of this assay is that the endpoint is objective and requires minimal experience by the investigator to perform. The burrowing assay has been validated for models of post-surgical pain in mice and in models of peripheral nerve injury, osteoarthritis and inflammation induced by CFA in rats (Jirkof et al., 2010; Andrews et al., 2012; Bryden et al., 2015). The assay is also capable of detecting side effects of analgesics, such as drowsiness, although it may be difficult to distinguish whether decreased burrowing arises from lack of efficacy or adverse effects unless additional behavioral tests or full dose-response curves are performed (Andrews et al., 2012). In addition to burrowing, other spontaneous behaviors can be assessed, including nesting construction and food hoarding (Deacon, 2006a; Rock et al., 2014).

### Weight Bearing and Gait Analysis

Gait and weight bearing of rodents can be analyzed as a surrogate measure of nociception and are typically considered measures of non-evoked or stimulus-independent “pain”. While weight bearing is typically considered to be a measure of non-stimulus evoked nociception, it can be argued that ambulation itself applies a nociceptive mechanical stimulus to the affected limb(s), and it may therefore be a measure of stimulus-evoked nociceptive behavior, especially in the dynamic weight bearing test or gait analysis tests.

Static weight bearing or incapacitance assays measure the distribution of weight across the hind paws and typically involve placing the animal in an inclined holder forcing placement of the hind paws on two independent pressure sensors (Figure 6C). Unequal weight distribution between the ipsilateral and contralateral paw are interpreted as a natural adjustment to the degree of nociception experienced, and have been observed in a number of models including monosodium iodoacetate (MIA)-induced osteoarthritis, bone cancer-induced pain, carrageenan-induced inflammation and sciatic nerve crush injury (Schött et al., 1994; Medhurst et al., 2002; Bove et al., 2003; Buys and Alphonso, 2014). As the test is performed in relatively unrestrained rodents, it relies heavily on the animal freely taking up the correct stance, which can be difficult to achieve in mice.

An additional disadvantage of incapacitance or static weight bearing tests is that only models with unilateral hind paw nociception can be assessed in this manner. Accordingly, dynamic weight bearing or gait analysis may provide similar data without the need for extensive animal and experimenter training.

The Advanced Dynamic Weight Bearing apparatus (Bioseb) was developed as a modification from static weight bearing or incapacitance tests and computes weight bearing for each of the front and rear paws, weight ratio and paw surface area in freely moving animals (Griffioen et al., 2015). The dynamic weight-bearing test is able to detect reduced weight bearing behaviors of the affected hind limb in multiple pain models, including CFA-induced inflammation, chronic constriction injury, bone cancer pain and antigen-induced arthritis (Tetreault et al., 2011; Robinson et al., 2012; Quadros et al., 2015). Similarly, it overcomes some of the experimental difficulties of static weight bearing analysis, albeit no information on gait can be obtained.

Prior to the development of automated digitized platforms, this test was performed by coloring the animal’s paws with ink; the animal was then allowed to walk freely on a paper, which could be scanned for analysis (Ishikawa et al., 2014). This assay is based on the hypothesis that a rodent with spontaneous “pain” will guard the “painful” paw, leading to changes in its gait (exhibiting a limp or changes in stride size, for instance) in addition to changes in weight bearing (Jacobs et al., 2014).

Gait analysis in freely walking rodents is used to study changes in limb movement and positioning in models with sensori-motor dysfunction, including Parkinson’s disease, spinal cord injury and stroke. A large number of parameters can be analyzed, including paw intensity (a measure of paw pressure or weight bearing), paw print parameters (e.g., toe spread, print length, print width, print area), dynamic parameters (e.g., stance phase, swing phase, duty cycle, stride length, swing speed) and regularity index (a measure of interlimb coordination). Some of these parameters are altered in rodent models of pain, making gait analysis a method that is increasingly used to quantify non-stimulus evoked or spontaneous nociception in rodents.

Several commercial automated gait analysis systems have been developed, including the CatWalk XT (Noldus) and GaitLab (ViewPoint Behavior Technology), which use internally reflected light to illuminate paw prints as an animal walks across an elevated glass floor (Figure 6D), and DigiGait (Mouse Specifics Inc.) and GaitScan/TreadScan (CleverSys), which use video recordings and automated software to analyze paw prints of animals walking on a transparent belt treadmill or clear floor walkway (Berryman et al., 2009; Parvathy and Masocha, 2013; Adams et al., 2016). A disadvantage of systems that only use video recordings (e.g., DigiGait, GaitScan/TreadScan) is that they cannot measure paw print intensity or pressure (weight bearing parameters), which is relevant for pain models.

In unilateral pain models, many changes in gait parameters are observed, including reduced intensity (paw pressure), reduced print area, reduced stance phase duration (time spent on paw), increased swing phase duration (time spent off paw) in the ipsilateral hind paw compared to contralateral hind paw, consistent with reduced weight bearing and guarding behaviors (Parvathy and Masocha, 2013; Yin et al., 2016). As altered weight bearing can be a major symptom of human pain conditions, these tests were developed to improve translation of rodent nociception models to the clinic. However, it is unclear to what degree changes in gait in rodent models reflect altered “pain” or nociception, or conversely, anti-nociception or analgesia. For example, in models of burn pain, chemotherapy-induced neuropathy, and intra-articular carrageenan, changes in mechanical allodynia as measured by Von Frey and changes in gait parameters do not always correlate (Gabriel et al., 2009; Boehmerle et al., 2014; Yin et al., 2016; Deuis et al., 2017b). It remains to be determined whether pain behavior outcomes obtained using gait and weight-bearing analysis will translate more (or less) readily to the clinic compared to stimulus-evoked methods.

### Automated Behavioral Analysis

Analysis of behaviors in unrestrained animals using automated technologies is increasingly being used to study non-stimulus evoked pain in rodents. Behaviors that are analyzed include locomotive activity (still, walking, trotting, running), distance traveled, velocity, grooming, posture, eating/drinking and foraging. By comparing the frequencies of behaviors in animal models of pain as opposed to control states, inference about different “pain” states (and especially spontaneous nociception) can be made. A caveat is, however, that no “pain-specific” behaviors are captured, leading to potential interference from drug or phenotype effects that could mask nociception, or anti-nociception. As the animal is unaware of the researcher and neither needs to be restrained or trained, this technique eliminates operator subjectivity and reduces animal stress. Automated behavioral analysis can be performed in a dedicated apparatus (Behavioral Spectrometer, Behavioral Instruments) or in a home cage (HomeCageScan, CleverSys; PhenoTyper, Noldus), using automated video analysis, vibration sensors, photobeams, and combinations thereof.

The Behavioral Spectrometer (Behavioral Instruments) is purpose built apparatus consisting of an enclosed box (~30 × 30 × 30 cm) with a ceiling-mounted fish-eye lens, accelerometer and a row of wall-mounted photobeams (Figure 6E). The spectrometer is capable of recording 23 different types of behaviors in real time, including ambulation (still, walking, trotting, running), grooming behaviors, and rearing behaviors, as well as distance traveled and average velocity (Brodkin et al., 2014). The Behavioral Spectrometer has been validated in a mouse model of carrageenan-induced hind paw inflammation, where the frequency of grooming was increased and the number of ambulation’s were decreased (Brodkin et al., 2014).

The HomeCageScan (CleverSys) uses automated video analysis to classify 38 pre-defined behaviors of mice in a home cage, including walking, rearing, sniffing, stretching, jumping, digging, foraging, sleeping, eating, drinking, hanging and grooming as well as distance traveled (Roughan et al., 2009). The HomeCageScan has been validated in mouse models of post-surgical pain following vasectomy and laparotomy (Roughan et al., 2009, 2016).

## Conclusion

Pain is a multifaceted and diverse experience that can be categorized into a number of types and modalities, depending on the presentation and triggering stimulus of the pain event. An organism’s response to nociception and the subsequent antinociceptive treatment is likewise varied, creating a need for a reliable way to assess “pain” levels and prospective treatments despite this variation.

One way to evaluate nociception in non-communicating subjects is through observation of behavior. So far, no single behavioral assay can capture the full spectrum of nociception in non-communicating subjects. Accordingly, translation of research using experimental nociceptive assays to pain treatment in the clinic has met with some difficulties. Appreciation that the human pain experience encompasses multiple stimulus modalities, distinct molecular mechanisms and sensory, motor, vegetative, emotional, motivational components should highlight the need for carefully designed experiments that take this complexity into consideration. Although animal models of pain have undoubtedly provided key advances, the advantages and disadvantages of each model and behavioral test should be taken into account to obtain objective and meaningful results that will improve our understanding and management of pain.

## Author Contributions

JRD, LSD and IV contributed to the preparation, revision and approval of the final manuscript.

## Conflict of Interest Statement

The authors declare that the research was conducted in the absence of any commercial or financial relationships that could be construed as a potential conflict of interest.

## References

[B1] Adams B. L. Guo W. Gors R. T. Knopp K. L. (2016). Pharmacological interrogation of a rodent forced ambulation model: leveraging gait impairment as a measure of pain behavior pre-clinically. Osteoarthr. Cartil. 24, 1928–1939. 10.1016/j.joca.2016.05.022 27450884

[B2] Allchorne A. J. Broom D. C. Woolf C. J. (2005). Detection of cold pain, cold allodynia and cold hyperalgesia in freely behaving rats. Mol. Pain 1:36. 10.1186/1744-8069-1-36 16354295 PMC1325266

[B3] Andrews N. Legg E. Lisak D. Issop Y. Richardson D. Harper S.. (2012). Spontaneous burrowing behaviour in the rat is reduced by peripheral nerve injury or inflammation associated pain. Eur. J. Pain 16, 485–495. 10.1016/j.ejpain.2011.07.012 22396078

[B4] Ankier S. I. (1974). New hot plate tests to quantify antinociceptive and narcotic antagonist activities. Eur. J. Pharmacol. 27, 1–4. 10.1016/0014-2999(74)90195-2 4853341

[B5] Anseloni V. C. Ennis M. Lidow M. S. (2003). Optimization of the mechanical nociceptive threshold testing with the Randall-Selitto assay. J. Neurosci. Methods 131, 93–97. 10.1016/s0165-0270(03)00241-3 14659828

[B6] Balayssac D. Ling B. Ferrier J. Pereira B. Eschalier A. Authier N. (2014). Assessment of thermal sensitivity in rats using the thermal place preference test: description and application in the study of oxaliplatin-induced acute thermal hypersensitivity and inflammatory pain models. Behav. Pharmacol. 25, 99–111. 10.1097/fbp.0000000000000026 24525711

[B150] Banik R. K. Kabadi R. A. (2013). A modified Hargreaves’ method for assessing threshold temperatures for heat nociception. J. Neurosci. Methods 219, 41–51. 10.1016/j.jneumeth.2013.06.005 23796910 PMC3759573

[B7] Baron R. Binder A. Wasner G. (2010). Neuropathic pain: diagnosis, pathophysiological mechanisms, and treatment. Lancet Neurol. 9, 807–819. 10.1016/S1474-4422(10)70143-5 20650402

[B8] Bautista D. M. Siemens J. Glazer J. M. Tsuruda P. R. Basbaum A. I. Stucky C. L.. (2007). The menthol receptor TRPM8 is the principal detector of environmental cold. Nature 448, 204–208. 10.1038/nature05910 17538622

[B9] Bennett D. L. Woods C. G. (2014). Painful and painless channelopathies. Lancet Neurol. 13, 587–599. 10.1016/s1474-4422(14)70024-9 24813307

[B10] Berge O. G. Garcia-Cabrera I. Hole K. (1988). Response latencies in the tail-flick test depend on tail skin temperature. Neurosci. Lett. 86, 284–288. 10.1016/0304-3940(88)90497-1 3380319

[B11] Berryman E. R. Harris R. L. Moalli M. Bagi C. M. (2009). Digigait quantitation of gait dynamics in rat rheumatoid arthritis model. J. Musculoskelet. Neuronal Interact. 9, 89–98. 19516084

[B12] Boehmerle W. Huehnchen P. Peruzzaro S. Balkaya M. Endres M. (2014). Electrophysiological, behavioral and histological characterization of paclitaxel, cisplatin, vincristine and bortezomib-induced neuropathy in C57Bl/6 mice. Sci. Rep. 4:6370. 10.1038/srep06370 25231679 PMC5377307

[B13] Bove S. E. Calcaterra S. L. Brooker R. M. Huber C. M. Guzman R. E. Juneau P. L.. (2003). Weight bearing as a measure of disease progression and efficacy of anti-inflammatory compounds in a model of monosodium iodoacetate-induced osteoarthritis. Osteoarthr. Cartil. 11, 821–830. 10.1016/s1063-4584(03)00163-8 14609535

[B14] Bradman M. J. Ferrini F. Salio C. Merighi A. (2015). Practical mechanical threshold estimation in rodents using von Frey hairs/Semmes-Weinstein monofilaments: towards a rational method. J. Neurosci. Methods 255, 92–103. 10.1016/j.jneumeth.2015.08.010 26296284

[B15] Brenner D. S. Golden J. P. Gereau R. W. IV (2012). A novel behavioral assay for measuring cold sensation in mice. PLoS One 7:e39765. 10.1371/journal.pone.0039765 22745825 PMC3382130

[B16] Brenner D. S. Golden J. P. Vogt S. K. Gereau R. W. (2015). A simple and inexpensive method for determining cold sensitivity and adaptation in mice. J. Vis. Exp. 97:e52640. 10.3791/52640 25867969 PMC4401362

[B17] Brodkin J. Frank D. Grippo R. Hausfater M. Gulinello M. Achterholt N.. (2014). Validation and implementation of a novel high-throughput behavioral phenotyping instrument for mice. J. Neurosci. Methods 224, 48–57. 10.1016/j.jneumeth.2013.12.010 24384067 PMC4305388

[B18] Bryden L. A. Nicholson J. R. Doods H. Pekcec A. (2015). Deficits in spontaneous burrowing behavior in the rat bilateral monosodium iodoacetate model of osteoarthritis: an objective measure of pain-related behavior and analgesic efficacy. Osteoarthr. Cartil. 23, 1605–1612. 10.1016/j.joca.2015.05.001 25966657

[B19] Buys M. J. Alphonso C. (2014). Novel use of perineural pregabalin infusion for analgesia in a rat neuropathic pain model. Anesth. Analg. 119, 481–488. 10.1213/ane.0000000000000291 24914629

[B20] Carlton S. M. Lekan H. A. Kim S. H. Chung J. M. (1994). Behavioral manifestations of an experimental model for peripheral neuropathy produced by spinal nerve ligation in the primate. Pain 56, 155–166. 10.1016/0304-3959(94)90090-6 8008406

[B21] Carter R. B. (1991). Differentiating analgesic and non-analgesic drug activities on rat hot plate: effect of behavioral endpoint. Pain 47, 211–220. 10.1016/0304-3959(91)90207-e 1762817

[B22] Chaplan S. R. Bach F. W. Pogrel J. W. Chung J. M. Yaksh T. L. (1994). Quantitative assessment of tactile allodynia in the rat paw. J. Neurosci. Methods 53, 55–63. 10.1016/0165-0270(94)90144-9 7990513

[B23] Chapman C. R. Casey K. L. Dubner R. Foley K. M. Gracely R. H. Reading A. E. (1985). Pain measurement: an overview. Pain 22, 1–31. 10.1016/0304-3959(85)90145-9 4011282

[B24] Choi Y. Yoon Y. W. Na H. S. Kim S. H. Chung J. M. (1994). Behavioral signs of ongoing pain and cold allodynia in a rat model of neuropathic pain. Pain 59, 369–376. 10.1016/0304-3959(94)90023-x 7708411

[B25] Colburn R. W. Lubin M. L. Stone D. J. Jr. Wang Y. Lawrence D. D’Andrea M. R.. (2007). Attenuated cold sensitivity in TRPM8 null mice. Neuron 54, 379–386. 10.1016/j.neuron.2007.04.017 17481392

[B26] Coutaux A. Adam F. Willer J. C. Le Bars D. (2005). Hyperalgesia and allodynia: peripheral mechanisms. Joint Bone Spine 72, 359–371. 10.1016/j.jbspin.2004.01.010 16214069

[B27] Cox J. J. Reimann F. Nicholas A. K. Thornton G. Roberts E. Springell K.. (2006). An SCN9A channelopathy causes congenital inability to experience pain. Nature 444, 894–898. 10.1038/nature05413 17167479 PMC7212082

[B28] Cruz-Almeida Y. Fillingim R. B. (2014). Can quantitative sensory testing move us closer to mechanism-based pain management? Pain Med. 15, 61–72. 10.1111/pme.12230 24010588 PMC3947088

[B29] Dalla Costa E. Stucke D. Dai F. Minero M. Leach M. C. Lebelt D. (2016). Using the horse grimace scale (HGS) to assess pain associated with acute laminitis in horses *(Equus caballus)*. Animals 6:47. 10.3390/ani6080047 27527224 PMC4997272

[B30] D’Amour F. E. Smith D. L. (1941). A method for determining loss of pain sensation. J. Pharmacol. Exp. Ther. 72, 74–79.

[B31] Deacon R. M. (2006a). Assessing hoarding in mice. Nat. Protoc. 1, 2828–2830. 10.1038/nprot.2006.171 17406541

[B32] Deacon R. M. (2006b). Burrowing in rodents: a sensitive method for detecting behavioral dysfunction. Nat. Protoc. 1, 118–121. 10.1038/nprot.2006.19 17406222

[B33] Decosterd I. Woolf C. J. (2000). Spared nerve injury: an animal model of persistent peripheral neuropathic pain. Pain 87, 149–158. 10.1016/s0304-3959(00)00276-1 10924808

[B34] Defrin R. Ohry A. Blumen N. Urca G. (2002). Sensory determinants of thermal pain. Brain 125, 501–510. 10.1093/brain/awf055 11872608

[B35] Defrin R. Shachal-Shiffer M. Hadgadg M. Peretz C. (2006). Quantitative somatosensory testing of warm and heat-pain thresholds: the effect of body region and testing method. Clin. J. Pain 22, 130–136. 10.1097/01.ajp.0000154048.68273.d8 16428946

[B36] Dell R. B. Holleran S. Ramakrishnan R. (2002). Sample size determination. ILAR J. 43, 207–213. 10.1093/ilar.43.4.207 12391396 PMC3275906

[B38] Deuis J. R. Dekan Z. Wingerd J. S. Smith J. J. Munasinghe N. R. Bhola R. F.. (2017a). Pharmacological characterisation of the highly Na_V_1.7 selective spider venom peptide Pn3a. Sci. Rep. 7:40883. 10.1038/srep40883 28106092 PMC5247677

[B41] Deuis J. R. Yin K. Cooper M. A. Schroder K. Vetter I. (2017b). Role of the NLRP3 inflammasome in a model of acute burn-induced pain. Burns 43, 304–309. 10.1016/j.burns.2016.09.001 28040362

[B39] Deuis J. R. Lim Y. L. Rodrigues de Sousa S. Lewis R. J. Alewood P. F. Cabot P. J.. (2014). Analgesic effects of clinically used compounds in novel mouse models of polyneuropathy induced by oxaliplatin and cisplatin. Neuro Oncol. 16, 1324–1332. 10.1093/neuonc/nou048 24714523 PMC4165414

[B37] Deuis J. R. Vetter I. (2016). The thermal probe test: a novel behavioral assay to quantify thermal paw withdrawal thresholds in mice. Temperature 3, 199–207. 10.1080/23328940.2016.1157668 27857950 PMC4965000

[B40] Deuis J. R. Whately E. Brust A. Inserra M. C. Asvadi N. H. Lewis R. J.. (2015). Activation of κ opioid receptors in cutaneous nerve endings by conorphin-1, a novel subtype-selective conopeptide, does not mediate peripheral analgesia. ACS Chem. Neurosci. 6, 1751–1758. 10.1021/acschemneuro.5b00113 26225903

[B42] Deuis J. R. Zimmermann K. Romanovsky A. A. Possani L. D. Cabot P. J. Lewis R. J.. (2013). An animal model of oxaliplatin-induced cold allodynia reveals a crucial role for Na_v_1.6 in peripheral pain pathways. Pain 154, 1749–1757. 10.1016/j.pain.2013.05.032 23711479 PMC3748219

[B43] Dixon W. J. (1980). Efficient analysis of experimental observations. Annu. Rev. Pharmacol. Toxicol. 20, 441–462. 10.1146/annurev.pa.20.040180.002301 7387124

[B44] Dubin A. E. Patapoutian A. (2010). Nociceptors: the sensors of the pain pathway. J. Clin. Invest. 120, 3760–3772. 10.1172/jci42843 21041958 PMC2964977

[B45] Dubner R. (1983). Pain research in animals. Ann. N Y Acad. Sci. 406, 128–132. 10.1111/j.1749-6632.1983.tb53494.x 6410957

[B46] Espejo E. F. Mir D. (1993). Structure of the rat’s behaviour in the hot plate test. Behav. Brain Res. 56, 171–176. 10.1016/0166-4328(93)90035-o 8240711

[B47] Festing M. F. Altman D. G. (2002). Guidelines for the design and statistical analysis of experiments using laboratory animals. ILAR J. 43, 244–258. 10.1093/ilar.43.4.244 12391400

[B48] Gabriel A. F. Marcus M. A. Walenkamp G. H. Joosten E. A. (2009). The CatWalk method: assessment of mechanical allodynia in experimental chronic pain. Behav. Brain Res. 198, 477–480. 10.1016/j.bbr.2008.12.018 19146883

[B49] Gamble G. D. Milne R. J. (1989). Repeated exposure to sham testing procedures reduces reflex withdrawal and hot-plate latencies: attenuation of tonic descending inhibition? Neurosci. Lett. 96, 312–317. 10.1016/0304-3940(89)90397-2 2717057

[B50] Gaston-Johansson F. Albert M. Fagan E. Zimmerman L. (1990). Similarities in pain descriptions of four different ethnic-culture groups. J. Pain Symptom Manage. 5, 94–100. 10.1016/s0885-3924(05)80022-3 2348093

[B51] Giglio C. A. Defino H. L. da-Silva C. A. de-Souza A. S. Del Bel E. A. (2006). Behavioral and physiological methods for early quantitative assessment of spinal cord injury and prognosis in rats. Braz. J. Med. Biol. Res. 39, 1613–1623. 10.1590/s0100-879x2006001200013 17160271

[B52] Gregory N. S. Harris A. L. Robinson C. R. Dougherty P. M. Fuchs P. N. Sluka K. A. (2013). An overview of animal models of pain: disease models and outcome measures. J. Pain 14, 1255–1269. 10.1016/j.jpain.2013.06.008 24035349 PMC3818391

[B53] Griffioen M. A. Dernetz V. H. Yang G. S. Griffith K. A. Dorsey S. G. Renn C. L. (2015). Evaluation of dynamic weight bearing for measuring nonevoked inflammatory hyperalgesia in mice. Nurs. Res. 64, 81–87. 10.1097/NNR.0000000000000082 25738619 PMC4351786

[B54] Gritsch S. Bali K. K. Kuner R. Vardeh D. (2016). Functional characterization of a mouse model for central post-stroke pain. Mol. Pain 12:1744806916629049. 10.1177/1744806916629049 27030713 PMC4956143

[B55] Guilford B. L. Ryals J. M. Wright D. E. (2011). Phenotypic changes in diabetic neuropathy induced by a high-fat diet in diabetic C57BL/6 mice. Exp. Diabetes Res. 2011:848307. 10.1155/2011/848307 22144990 PMC3226416

[B56] Hager C. Biernot S. Buettner M. Glage S. Keubler L. M. Held N.. (2017). The sheep grimace scale as an indicator of post-operative distress and pain in laboratory sheep. PLoS One 12:e0175839. 10.1371/journal.pone.0175839 28422994 PMC5396914

[B57] Han J. S. Bird G. C. Li W. Jones J. Neugebauer V. (2005). Computerized analysis of audible and ultrasonic vocalizations of rats as a standardized measure of pain-related behavior. J. Neurosci. Methods 141, 261–269. 10.1016/j.jneumeth.2004.07.005 15661308

[B58] Hargreaves K. Dubner R. Brown F. Flores C. Joris J. (1988). A new and sensitive method for measuring thermal nociception in cutaneous hyperalgesia. Pain 32, 77–88. 10.1016/0304-3959(88)90026-7 3340425

[B59] Harvey V. L. Dickenson A. H. (2009). Behavioural and electrophysiological characterisation of experimentally induced osteoarthritis and neuropathy in C57Bl/6 mice. Mol. Pain 5:18. 10.1186/1744-8069-5-18 19379487 PMC2678995

[B60] Hirst J. A. Howick J. Aronson J. K. Roberts N. Perera R. Koshiaris C.. (2014). The need for randomization in animal trials: an overview of systematic reviews. PLoS One 9:e98856. 10.1371/journal.pone.0098856 24906117 PMC4048216

[B61] Huang S. M. Li X. Yu Y. Wang J. Caterina M. J. (2011). TRPV3 and TRPV4 ion channels are not major contributors to mouse heat sensation. Mol. Pain 7:37. 10.1186/1744-8069-7-37 21586160 PMC3123222

[B62] Hunskaar S. Fasmer O. B. Hole K. (1985). Acetylsalicylic acid, paracetamol and morphine inhibit behavioral responses to intrathecally administered substance P or capsaicin. Life Sci. 37, 1835–1841. 10.1016/0024-3205(85)90227-9 2414631

[B63] Irwin S. Houde R. W. Bennett D. R. Hendershot L. C. Seevers M. H. (1951). The effects of morphine methadone and meperidine on some reflex responses of spinal animals to nociceptive stimulation. J. Pharmacol. Exp. Ther. 101, 132–143. 14814606

[B64] Ishikawa G. Nagakura Y. Takeshita N. Shimizu Y. (2014). Efficacy of drugs with different mechanisms of action in relieving spontaneous pain at rest and during movement in a rat model of osteoarthritis. Eur. J. Pharmacol. 738, 111–117. 10.1016/j.ejphar.2014.05.048 24939049

[B65] Jacobs B. Y. Kloefkorn H. E. Allen K. D. (2014). Gait analysis methods for rodent models of osteoarthritis. Curr. Pain Headache Rep. 18:456. 10.1007/s11916-014-0456-x 25160712 PMC4180257

[B66] Jensen T. S. Finnerup N. B. (2014). Allodynia and hyperalgesia in neuropathic pain: clinical manifestations and mechanisms. Lancet Neurol. 13, 924–935. 10.1016/s1474-4422(14)70102-4 25142459

[B68] Jensen T. S. Gottrup H. Sindrup S. H. Bach F. W. (2001). The clinical picture of neuropathic pain. Eur. J. Pharmacol. 429, 1–11. 10.1016/S0014-2999(01)01302-4 11698022

[B67] Jensen T. S. Yaksh T. L. (1986). Comparison of the antinociceptive action of μ and Δ opioid receptor ligands in the periaqueductal gray matter, medial and paramedial ventral medulla in the rat as studied by the microinjection technique. Brain Res. 372, 301–312. 10.1016/0006-8993(86)91138-8 2871901

[B69] Jirkof P. Cesarovic N. Rettich A. Nicholls F. Seifert B. Arras M. (2010). Burrowing behavior as an indicator of post-laparotomy pain in mice. Front. Behav. Neurosci. 4:165. 10.3389/fnbeh.2010.00165 21031028 PMC2965018

[B70] Kayser V. Christensen D. (2000). Antinociceptive effect of systemic gabapentin in mononeuropathic rats, depends on stimulus characteristics and level of test integration. Pain 88, 53–60. 10.1016/s0304-3959(00)00307-9 11098099

[B71] Kim S. H. Chung J. M. (1992). An experimental model for peripheral neuropathy produced by segmental spinal nerve ligation in the rat. Pain 50, 355–363. 10.1016/0304-3959(92)90041-9 1333581

[B72] Knowlton W. M. Bifolck-Fisher A. Bautista D. M. McKemy D. D. (2010). TRPM8, but not TRPA1, is required for neural and behavioral responses to acute noxious cold temperatures and cold-mimetics *in vivo*. Pain 150, 340–350. 10.1016/j.pain.2010.05.021 20542379 PMC2897947

[B73] Langford D. J. Bailey A. L. Chanda M. L. Clarke S. E. Drummond T. E. Echols S.. (2010). Coding of facial expressions of pain in the laboratory mouse. Nat. Methods 7, 447–449. 10.1038/nmeth.1455 20453868

[B74] Leith J. L. Koutsikou S. Lumb B. M. Apps R. (2010). Spinal processing of noxious and innocuous cold information: differential modulation by the periaqueductal gray. J. Neurosci. 30, 4933–4942. 10.1523/JNEUROSCI.0122-10.2010 20371814 PMC6632802

[B75] Lötsch J. Dimova V. Lieb I. Zimmermann M. Oertel B. G. Ultsch A. (2015). Multimodal distribution of human cold pain thresholds. PLoS One 10:e0125822. 10.1371/journal.pone.0125822 25992576 PMC4439151

[B76] Lu R. Schmidtko A. (2013). Direct intrathecal drug delivery in mice for detecting *in vivo* effects of cGMP on pain processing. Methods Mol. Biol. 1020, 215–221. 10.1007/978-1-62703-459-3_14 23709036

[B77] Matsumiya L. C. Sorge R. E. Sotocinal S. G. Tabaka J. M. Wieskopf J. S. Zaloum A.. (2012). Using the Mouse Grimace Scale to reevaluate the efficacy of postoperative analgesics in laboratory mice. J. Am. Assoc. Lab. Anim. Sci. 51, 42–49. 22330867 PMC3276965

[B78] McGrath J. C. Drummond G. B. McLachlan E. M. Kilkenny C. Wainwright C. L. (2010). Guidelines for reporting experiments involving animals: the ARRIVE guidelines. Br. J. Pharmacol. 160, 1573–1576. 10.1111/j.1476-5381.2010.00873.x 20649560 PMC2936829

[B79] Medhurst S. J. Walker K. Bowes M. Kidd B. L. Glatt M. Muller M.. (2002). A rat model of bone cancer pain. Pain 96, 129–140. 10.1016/S0304-3959(01)00437-7 11932069

[B80] Menéndez L. Lastra A. Hidalgo A. Baamonde A. (2002). Unilateral hot plate test: a simple and sensitive method for detecting central and peripheral hyperalgesia in mice. J. Neurosci. Methods 113, 91–97. 10.1016/s0165-0270(01)00483-6 11741726

[B81] Millecamps M. Jourdan D. Leger S. Etienne M. Eschalier A. Ardid D. (2005). Circadian pattern of spontaneous behavior in monarthritic rats: a novel global approach to evaluation of chronic pain and treatment effectiveness. Arthritis Rheum. 52, 3470–3478. 10.1002/art.21403 16258901

[B82] Miller A. L. Leach M. C. (2015). Using the mouse grimace scale to assess pain associated with routine ear notching and the effect of analgesia in laboratory mice. Lab. Anim. 49, 117–120. 10.1177/0023677214559084 25378137

[B83] Minett M. S. Eijkelkamp N. Wood J. N. (2014). Significant determinants of mouse pain behaviour. PLoS One 9:e104458. 10.1371/journal.pone.0104458 25101983 PMC4125188

[B84] Minett M. S. Quick K. Wood J. N. (2011). Behavioral measures of pain thresholds. Curr. Protoc. Mouse Biol. 1, 383–412. 10.1002/9780470942390.mo110116 26068997

[B85] Mogil J. S. (2009). Animal models of pain: progress and challenges. Nat. Rev. Neurosci. 10, 283–294. 10.1038/nrn2606 19259101

[B86] Moqrich A. Hwang S. W. Earley T. J. Petrus M. J. Murray A. N. Spencer K. S.. (2005). Impaired thermosensation in mice lacking TRPV3, a heat and camphor sensor in the skin. Science 307, 1468–1472. 10.1126/science.1108609 15746429

[B87] Morgan D. Mitzelfelt J. D. Koerper L. M. Carter C. S. (2012). Effects of morphine on thermal sensitivity in adult and aged rats. J. Gerontol. A Biol. Sci. Med. Sci. 67, 705–713. 10.1093/gerona/glr210 22193548 PMC3391067

[B88] O’Brien D. E. Brenner D. S. Gutmann D. H. Gereau R. W. IV (2013). Assessment of pain and itch behavior in a mouse model of neurofibromatosis type 1. J. Pain 14, 628–637. 10.1016/j.jpain.2013.01.770 23578956 PMC3672240

[B89] Ogren S. O. Berge O. G. (1984). Test-dependent variations in the antinociceptive effect of p-chloroamphetamine-induced release of 5-hydroxytryptamine. Neuropharmacology 23, 915–924. 10.1016/0028-3908(84)90005-4 6237274

[B90] Parvathy S. S. Masocha W. (2013). Gait analysis of C57BL/6 mice with complete Freund’s adjuvant-induced arthritis using the CatWalk system. BMC Musculoskelet. Disord. 14:14. 10.1186/1471-2474-14-14 23297850 PMC3608084

[B91] Pertovaara A. Kauppila T. Hämäläinen M. M. (1996). Influence of skin temperature on heat pain threshold in humans. Exp. Brain Res. 107, 497–503. 10.1007/bf00230429 8821389

[B92] Petrus M. Peier A. M. Bandell M. Hwang S. W. Huynh T. Olney N.. (2007). A role of TRPA1 in mechanical hyperalgesia is revealed by pharmacological inhibition. Mol. Pain 3:40. 10.1186/1744-8069-3-40 18086313 PMC2222610

[B93] Pitcher G. M. Ritchie J. Henry J. L. (1999). Paw withdrawal threshold in the von Frey hair test is influenced by the surface on which the rat stands. J. Neurosci. Methods 87, 185–193. 10.1016/s0165-0270(99)00004-7 11230815

[B94] Plone M. A. Emerich D. F. Lindner M. D. (1996). Individual differences in the hotplate test and effects of habituation on sensitivity to morphine. Pain 66, 265–270. 10.1016/0304-3959(96)03048-5 8880849

[B95] Quadros A. U. Pinto L. G. Fonseca M. M. Kusuda R. Cunha F. Q. Cunha T. M. (2015). Dynamic weight bearing is an efficient and predictable method for evaluation of arthritic nociception and its pathophysiological mechanisms in mice. Sci. Rep. 5:14648. 10.1038/srep14648 26511791 PMC4625149

[B96] Randall L. O. Selitto J. J. (1957). A method for measurement of analgesic activity on inflamed tissue. Arch. Int. Pharmacodyn. Ther. 111, 409–419. 13471093

[B97] Robinson I. Sargent B. Hatcher J. P. (2012). Use of dynamic weight bearing as a novel end-point for the assessment of Freund’s Complete Adjuvant induced hypersensitivity in mice. Neurosci. Lett. 524, 107–110. 10.1016/j.neulet.2012.07.017 22819976

[B98] Rock M. L. Karas A. Z. Rodriguez K. B. Gallo M. S. Pritchett-Corning K. Karas R. H.. (2014). The time-to-integrate-to-nest test as an indicator of wellbeing in laboratory mice. J. Am. Assoc. Lab. Anim. Sci. 53, 24–28. 24411776 PMC3894644

[B99] Rolke R. Baron R. Maier C. Tölle T. R. Treede R. D. Beyer A.. (2006). Quantitative sensory testing in the german research network on neuropathic pain (DFNS): standardized protocol and reference values. Pain 123, 231–243. 10.1016/j.pain.2006.01.041 16697110

[B100] Roughan J. V. Bertrand H. G. Isles H. M. (2016). Meloxicam prevents COX-2-mediated post-surgical inflammation but not pain following laparotomy in mice. Eur. J. Pain 20, 231–240. 10.1002/ejp.871 25908253 PMC4728739

[B101] Roughan J. V. Wright-Williams S. L. Flecknell P. A. (2009). Automated analysis of postoperative behaviour: assessment of HomeCageScan as a novel method to rapidly identify pain and analgesic effects in mice. Lab. Anim. 43, 17–26. 10.1258/la.2008.007156 19015177

[B102] Sandkühler J. (2009). Models and mechanisms of hyperalgesia and allodynia. Physiol. Rev. 89, 707–758. 10.1152/physrev.00025.2008 19342617

[B103] Santos-Nogueira E. Redondo Castro E. Mancuso R. Navarro X. (2012). Randall-Selitto test: a new approach for the detection of neuropathic pain after spinal cord injury. J. Neurotrauma 29, 898–904. 10.1089/neu.2010.1700 21682605 PMC3303094

[B104] Scholz J. Broom D. C. Youn D. H. Mills C. D. Kohno T. Suter M. R.. (2005). Blocking caspase activity prevents transsynaptic neuronal apoptosis and the loss of inhibition in lamina II of the dorsal horn after peripheral nerve injury. J. Neurosci. 25, 7317–7323. 10.1523/JNEUROSCI.1526-05.2005 16093381 PMC6725303

[B105] Schött E. Berge O. G. Angeby-Möller K. Hammarström G. Dalsgaard C. J. Brodin E. (1994). Weight bearing as an objective measure of arthritic pain in the rat. J. Pharmacol. Toxicol. Methods 31, 79–83. 10.1016/1056-8719(94)90046-9 8032098

[B106] Sotocinal S. G. Sorge R. E. Zaloum A. Tuttle A. H. Martin L. J. Wieskopf J. S.. (2011). The rat grimace scale: a partially automated method for quantifying pain in the laboratory rat via facial expressions. Mol. Pain 7:55. 10.1186/1744-8069-7-55 21801409 PMC3163602

[B107] Tappe-Theodor A. Kuner R. (2014). Studying ongoing and spontaneous pain in rodents—challenges and opportunities. Eur. J. Neurosci. 39, 1881–1890. 10.1111/ejn.12643 24888508

[B108] Tetreault P. Dansereau M. A. Doré-Savard L. Beaudet N. Sarret P. (2011). Weight bearing evaluation in inflammatory, neuropathic and cancer chronic pain in freely moving rats. Physiol. Behav. 104, 495–502. 10.1016/j.physbeh.2011.05.015 21620878

[B109] Tjolsen A. Rosland J. H. Berge O. G. Hole K. (1991). The increasing-temperature hot-plate test: an improved test of nociception in mice and rats. J. Pharmacol. Methods 25, 241–250. 10.1016/0160-5402(91)90014-v 2056753

[B110] Touska F. Winter Z. Mueller A. Vlachova V. Larsen J. Zimmermann K. (2016). Comprehensive thermal preference phenotyping in mice using a novel automated circular gradient assay. Temperature 3, 77–91. 10.1080/23328940.2015.1135689 27227099 PMC4861200

[B111] Vazquez G. Alvarez E. Navaza J. M. (1995). Surface tension of alchol water + water from 20 to 50 degrees celcius. J. Chem. Eng. Data 40, 611–614. 10.1021/je00019a016

[B112] Viscardi A. V. Hunniford M. Lawlis P. Leach M. Turner P. V. (2017). Development of a piglet grimace scale to evaluate piglet pain using facial expressions following castration and tail docking: a pilot study. Front. Vet. Sci. 4:51. 10.3389/fvets.2017.00051 28459052 PMC5394162

[B113] Vissers K. Meert T. (2005). A behavioral and pharmacological validation of the acetone spray test in gerbils with a chronic constriction injury. Anesth. Analg. 101, 457–464. 10.1213/01.ane.0000158471.41575.f0 16037162

[B114] Vriens J. Owsianik G. Hofmann T. Philipp S. E. Stab J. Chen X.. (2011). TRPM3 is a nociceptor channel involved in the detection of noxious heat. Neuron 70, 482–494. 10.1016/j.neuron.2011.02.051 21555074

[B115] Wallace V. C. Norbury T. A. Rice A. S. (2005). Ultrasound vocalisation by rodents does not correlate with behavioural measures of persistent pain. Eur. J. Pain 9, 445–452. 10.1016/j.ejpain.2004.10.006 15979025

[B116] Wibbenmeyer L. Sevier A. Liao J. Williams I. Latenser B. Lewis R.. (2011). Evaluation of the usefulness of two established pain assessment tools in a burn population. J. Burn Care Res. 32, 52–60. 10.1097/BCR.0b013e3182033359 21116190

[B117] Williams W. O. Riskin D. K. Mott A. K. (2008). Ultrasonic sound as an indicator of acute pain in laboratory mice. J. Am. Assoc. Lab. Anim. Sci. 47, 8–10. 18210991 PMC2652617

[B118] Winter C. A. Flataker L. (1965). Reaction thresholds to pressure in edematous hindpaws of rats and responses to analgesic drugs. J. Pharmacol. Exp. Ther. 150, 165–171. 5853697

[B119] Woolf C. J. Mannion R. J. (1999). Neuropathic pain: aetiology, symptoms, mechanisms, and management. Lancet 353, 1959–1964. 10.1016/s0140-6736(99)01307-0 10371588

[B120] Woolfe G. Macdonald A. D. (1944). The evaluation of the analgesic action of pethidine hydrocholoride (Demerol). J. Pharmacol. Exp. Ther. 80, 300–307.

[B121] Xing H. Chen M. Ling J. Tan W. Gu J. G. (2007). TRPM8 mechanism of cold allodynia after chronic nerve injury. J. Neurosci. 27, 13680–13690. 10.1523/JNEUROSCI.2203-07.2007 18077679 PMC6673615

[B122] Yalcin I. Charlet A. Freund-Mercier M. J. Barrot M. Poisbeau P. (2009). Differentiating thermal allodynia and hyperalgesia using dynamic hot and cold plate in rodents. J. Pain 10, 767–773. 10.1016/j.jpain.2009.01.325 19409860

[B123] Yamamoto K. Tsuboi M. Kambe T. Abe K. Nakatani Y. Kawakami K.. (2016). Oxaliplatin administration increases expression of the voltage-dependent calcium channel α2Δ-1 subunit in the rat spinal cord. J. Pharmacol. Sci. 130, 117–122. 10.1016/j.jphs.2016.01.006 26883453

[B126] Yeomans D. C. Pirec V. Proudfit H. K. (1996). Nociceptive responses to high and low rates of noxious cutaneous heating are mediated by different nociceptors in the rat: behavioral evidence. Pain 68, 133–140. 10.1016/s0304-3959(96)03176-4 9252008

[B124] Yeomans D. C. Proudfit H. K. (1994). Characterization of the foot withdrawal response to noxious radiant heat in the rat. Pain 59, 85–94. 10.1016/0304-3959(94)90051-5 7854807

[B125] Yeomans D. C. Proudfit H. K. (1996). Nociceptive responses to high and low rates of noxious cutaneous heating are mediated by different nociceptors in the rat: electrophysiological evidence. Pain 68, 141–150. 10.1016/s0304-3959(96)03177-6 9252009

[B127] Yin K. Deuis J. R. Lewis R. J. Vetter I. (2016). Transcriptomic and behavioural characterisation of a mouse model of burn pain identify the cholecystokinin 2 receptor as an analgesic target. Mol. Pain 12:1744806916665366. 10.1177/1744806916665366 27573516 PMC5007901

[B128] Yoburn B. C. Morales R. Kelly D. D. Inturrisi C. E. (1984). Constraints on the tailflick assay: morphine analgesia and tolerance are dependent upon locus of tail stimulation. Life Sci. 34, 1755–1762. 10.1016/0024-3205(84)90575-7 6727545

[B129] Zimmermann K. Deuis J. R. Inserra M. C. Collins L. S. Namer B. Cabot P. J.. (2013). Analgesic treatment of ciguatoxin-induced cold allodynia. Pain 154, 1999–2006. 10.1016/j.pain.2013.06.015 23778293

